# Chlorine-Induced Lung Injury From Hot Tub Exposure

**DOI:** 10.7759/cureus.70025

**Published:** 2024-09-23

**Authors:** Rafid Mustafa, Mohamed Gadallah, Alaeldin Elfaki, Bassey Asuquo

**Affiliations:** 1 Department of Internal Medicine, United Lincolnshire Hospitals NHS Trust, Boston, GBR; 2 Department of Respiratory Medicine, United Lincolnshire Hospitals NHS Trust, Boston, GBR; 3 Department of Chest Diseases, Alexandria University, Alexandria, EGY

**Keywords:** bronchiolitis obliterans, chlorine gas inhalation, hot tub lung, hypersensitivity pneumonitis (hp), mycobacterium avium-complex

## Abstract

Chlorine, a dense and irritating gas used in pool disinfection, can cause severe respiratory issues, including airway damage, alveolar injury, pulmonary edema, and chronic conditions such as bronchiolitis obliterans. This case study describes a patient initially thought to have hot tub lung (HTL) due to symptoms, hot tub use, and imaging findings. However, negative Mycobacterium tests and significant chlorine exposure led to a revised diagnosis of chlorine-induced lung injury. The diagnosis was further supported by the patient's clinical improvement and prior normal lung scans.

## Introduction

Chlorine, commonly used in pool disinfection, is a green-yellow gas with intermediate water solubility. Chlorine gas has an irritating, pungent odor (odor of bleach). It reacts with water to form toxic products: hydrochloric acid (HCL) (a caustic element) and hypochlorous acid (HOCl) (forms free radicals). It primarily impacts the lower respiratory tract. High levels of exposure can cause severe respiratory issues, including airway damage, alveolar injury, and pulmonary edema, while chronic exposure may lead to bronchiolitis obliterans, reactive airway dysfunction syndrome (RADS), and respiratory upper airway distress (RUDS) [1]. In contrast, hot tub lung (HTL) arises from inhaling aerosols contaminated with *Mycobacterium avium* complex (MAC) from poorly maintained hot tubs. HTL is characterized by a hypersensitivity reaction rather than an infection, with symptoms initially resembling flu and progressing to persistent respiratory problems. Diagnosing HTL involves a thorough evaluation of symptoms, imaging, exposure history, and microbiological testing to differentiate it from other respiratory conditions [2]. Here, we describe a case initially diagnosed as HTL that was later revised to chlorine-induced lung injury based on the patient’s history and investigations.

## Case presentation

A 65-year-old male presented with a six-month history of progressively worsening shortness of breath, accompanied by a cough and yellowish phlegm. He denied experiencing orthopnea, paroxysmal nocturnal dyspnea, chest pain, calf swelling, or redness. On admission, his vital signs were heart rate, 79 beats per minute; respiratory rate, 17 breaths per minute; temperature, 37°C; blood pressure, 133/78 mmHg; and type 1 respiratory failure (oxygen saturation of 89% corrected with 3 liters of oxygen). Physical examination revealed bilateral expiratory wheeze and faint basal crepitations, more prominent on the left side. Other systemic examinations were unremarkable. His past medical history included moderately differentiated adenocarcinoma of the rectum with low anterior resection performed seven years ago and reversal of ileostomy done five years ago. He had no prior history of respiratory conditions or gastroesophageal reflux disease (GERD).

**Figure 1:**
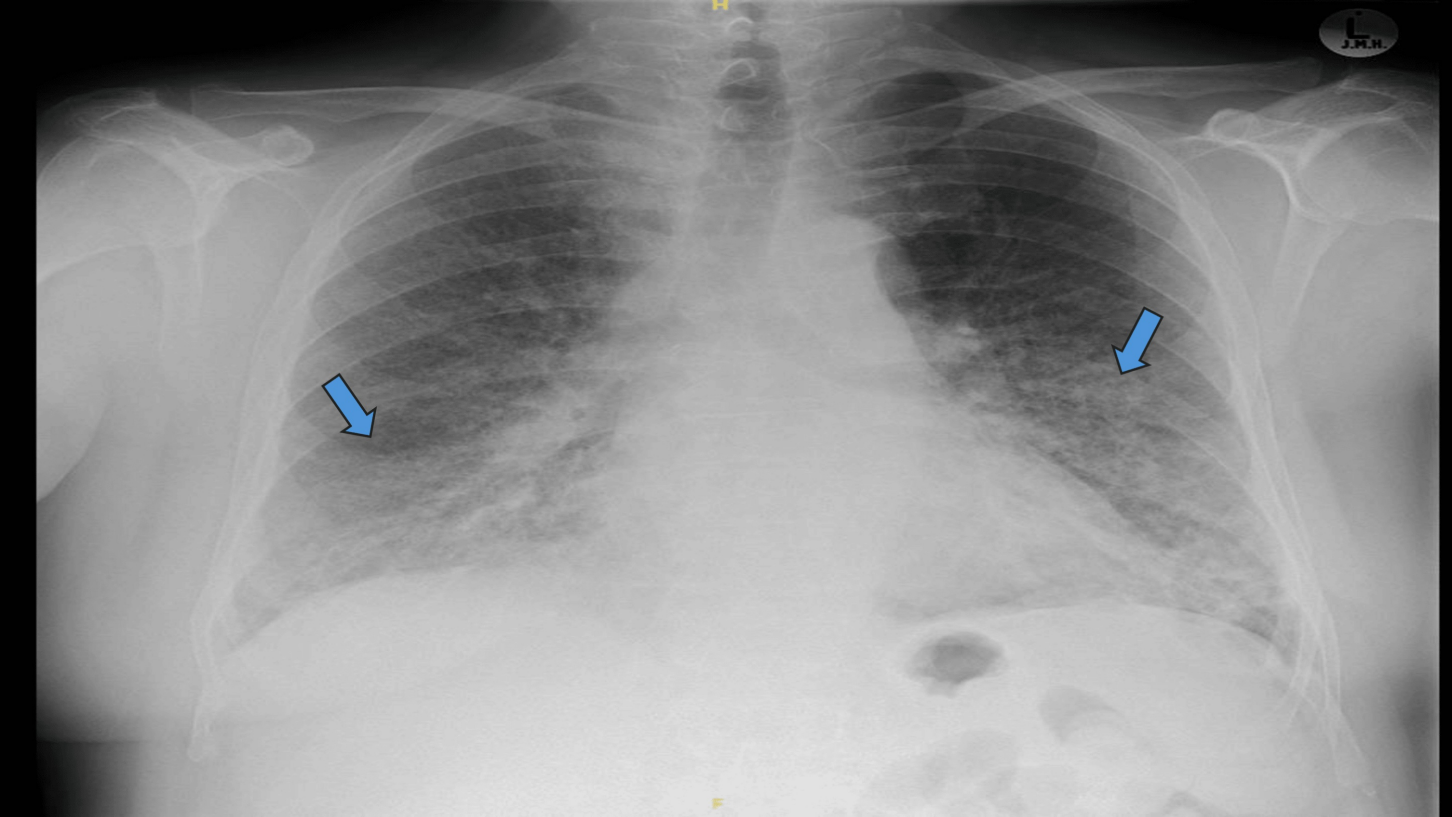
Chest X-ray from hospitalization 14 days prior to current admission Chest X-ray revealed patchy air space opacification in both the mid and lower lung zones, consistent with ongoing infections.

His medical history was notable for a hospitalization 14 days prior due to similar respiratory complaints. During that admission, blood tests showed elevated infective markers, and a chest X-ray revealed patchy air space opacification in both the mid and lower lung zones, consistent with ongoing infections (Figure 1). He was diagnosed with bilateral pneumonia and treated with intravenous antibiotics and oxygen therapy, resulting in clinical improvement and normalization of inflammatory markers. He was subsequently discharged with a five-day course of oral Levofloxacin.

**Figure 2:**
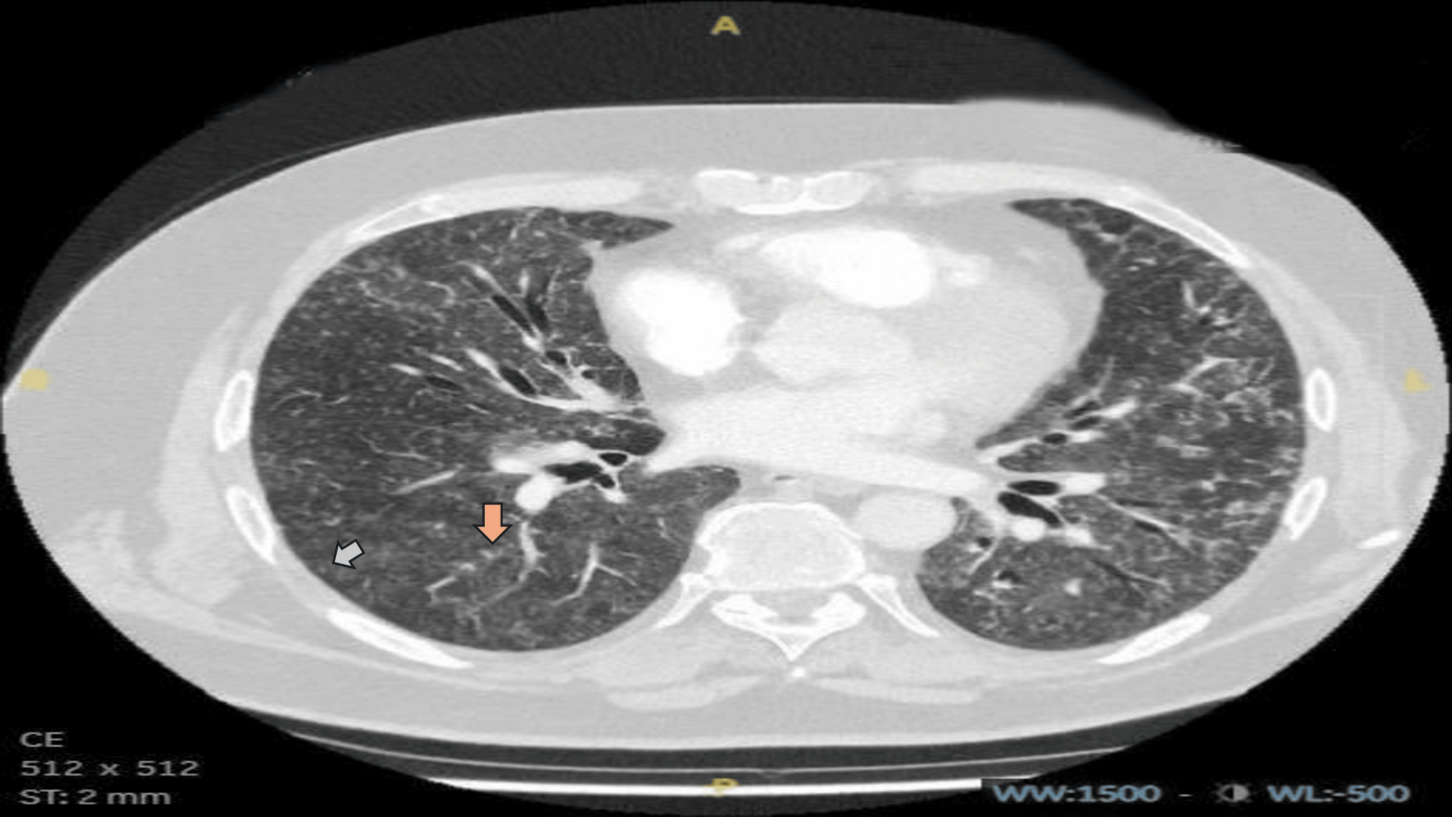
CTPA from current admission Initial CTPA revealed diffuse ground-glass opacities with a centrilobular nodular pattern in both lungs. Additional findings include mosaic attenuation, a tree-in-bud appearance, and areas of air trapping. The grey arrow indicates a centrilobular nodular pattern. The orange arrow indicates a tree in bud appearance. CTPA, computed tomography pulmonary angiography

**Figure 3:**
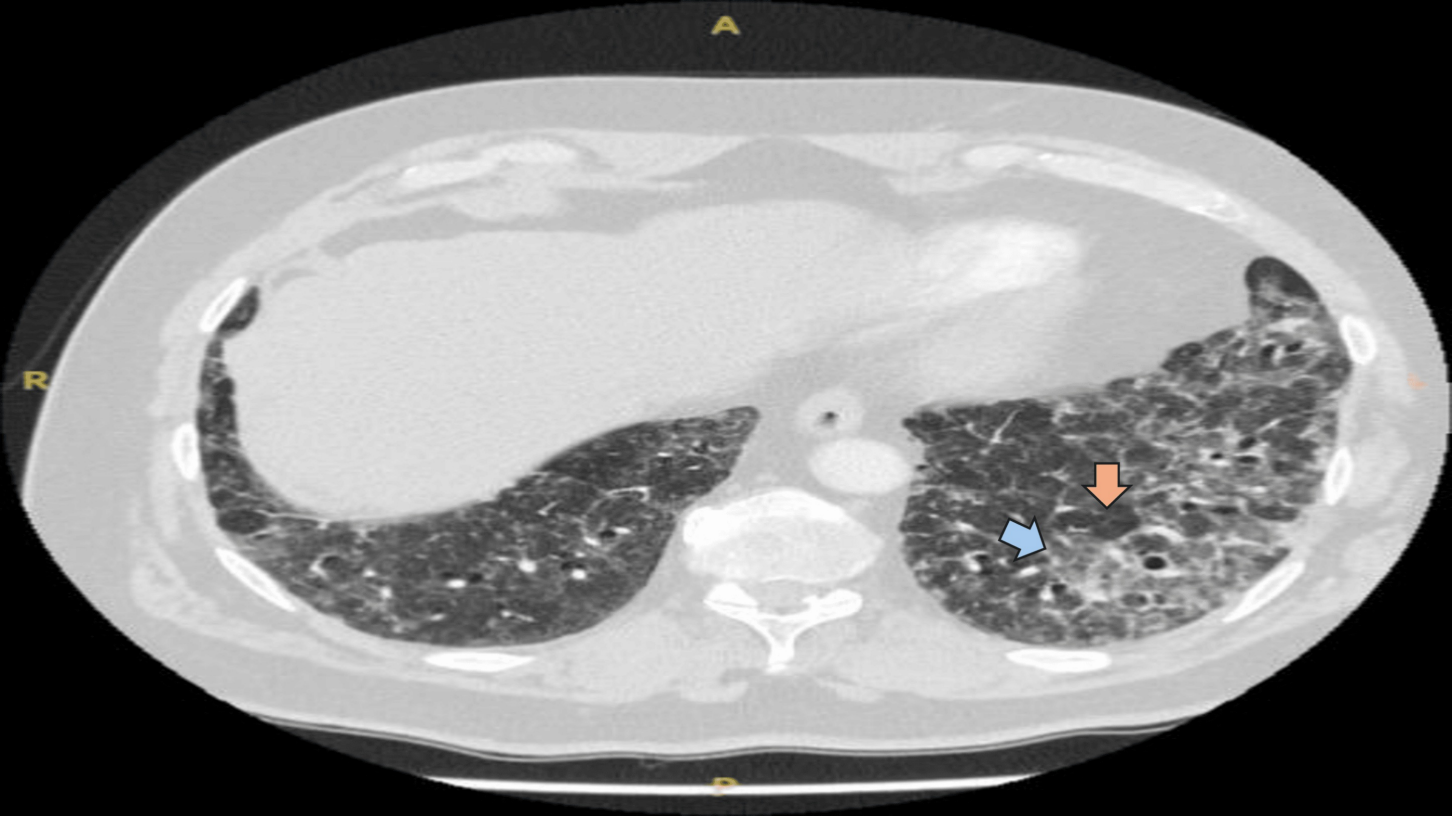
CTPA from current admission Initial CTPA revealing diffuse ground-glass opacities with a centrilobular nodular pattern in both lungs. Additional findings include mosaic attenuation, a tree-in-bud appearance, and areas of air trapping. The blue arrow indicates ground glass opacities. The orange arrow indicates areas of air trapping. CTPA, computed tomography pulmonary angiography

During his current admission, despite normal infective markers, a high D-dimer level of 1623 ng/mL (Table 1) raised suspicion of a pulmonary embolism (PE). A computed tomography pulmonary angiography (CTPA) was performed, which excluded PE but revealed diffuse ground-glass changes with a centrilobular nodular pattern in both lungs, along with mosaic attenuation and a tree-in-bud appearance (Figures 2, 3). These findings were consistent with hypersensitivity pneumonitis (HP), leading to a detailed inquiry into the patient’s exposure history.

**Table 1:** Blood test results during current admission CRP, C-reactive protein; WCC, white cell count; IgG, immunoglobulin G; ANA, anti-nuclear antibody; ENA, extractable nuclear antigen; RF, rheumatoid factor; CCP, cyclic citrullinated peptide

Test	Result	Range/Unit
CRP	10	0-5 mg/L
WCC	9.9	4.3-11.2x10^9^/L
Neutrophils	7.47	2.1-7.4x10^9^/L
D-dimer	1623	0-230 ng/mL
IgG to budgie serum/fea/dand	4	0-40 mg/L
IgG to pigeon serum/fea/drop	9	0-40 mg/L
IgG to parrot serum/fea/dand	4	0-40 mg/L
IgG to *Micropolyspora faeni*	4	0-60 mg/L
ANA	Negative	
Double-stranded DNA antibody	16.2	0-27 iu/mL
ENA antibody screen	Negative	
RF	20	0-14 IU/mL
IgG to *Aspergillus fumigatus*	37	0-40 mg/L
IgE to *Aspergillus fumigatus*	<0.35	0-0.35 kAU/L
*Haemophilus influenzae* antibody IgG	0.36	ug/Ml
CCP antibodies	8	0-17 U/mL
Angiotensin-converting enzyme	62	16-85 U/L
Glomerular basement membrane antibody	<2.9	0-20 U

Full history revealed that he is a non-smoker working in the sales department at a car showroom, with no exposure to dust, birds, or pets. He had installed a hot tub in an enclosed, unventilated room at home seven months ago, using it twice a week for two-hour sessions and disinfecting it with a high amount of stabilized chlorine granules. He was using three times the recommended amount. Based on his hot tub use and imaging results, the respiratory team initially suspected HTL. He was advised to stop using the hot tub and was prescribed oral prednisolone 40 mg once daily (OD). The extended sputum culture showed a light growth of yeast. Microscopy for acid alcohol fast bacilli (AAFB) was negative, and no Mycobacteria species were detected. Immunoglobulin profiles, autoantibodies, and screenings for *Aspergillus fumigatus*, *Micropolyspora faeni*, pigeon serum, and parrot serum were all negative (Table 1). Since he responded well to treatment, a lung biopsy and bronchoalveolar lavage (BAL) were not performed. He was discharged with a tapering dose of prednisolone, starting at 40 mg daily for seven days and then decreasing by 5 mg weekly until completion. Follow-up included pulmonary function tests in two months and a repeat CT scan in three months.

**Figure 4:**
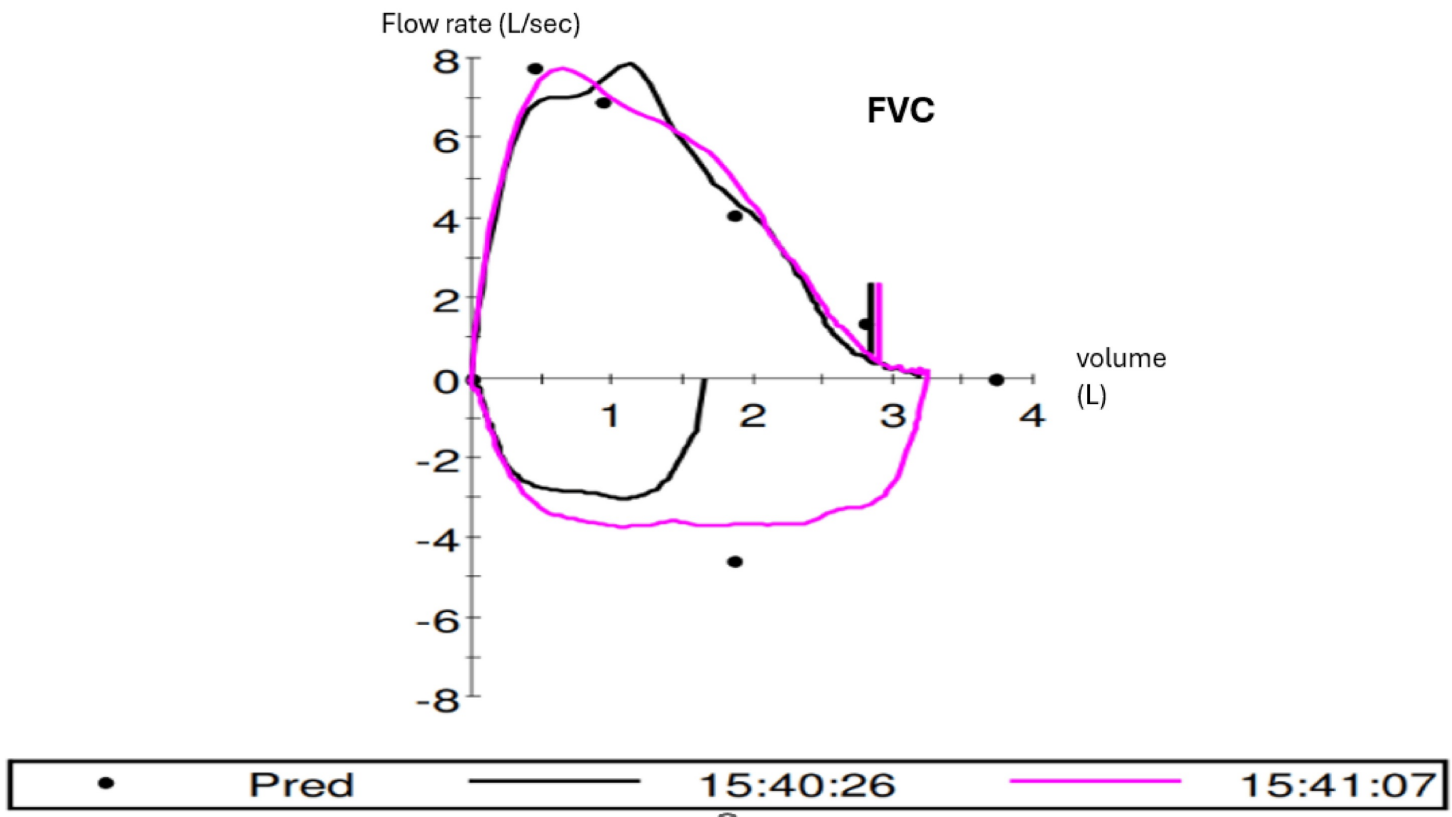
Spirometry of the patient FVC, forced vital capacity; Pred, predicted value

**Figure 5:**
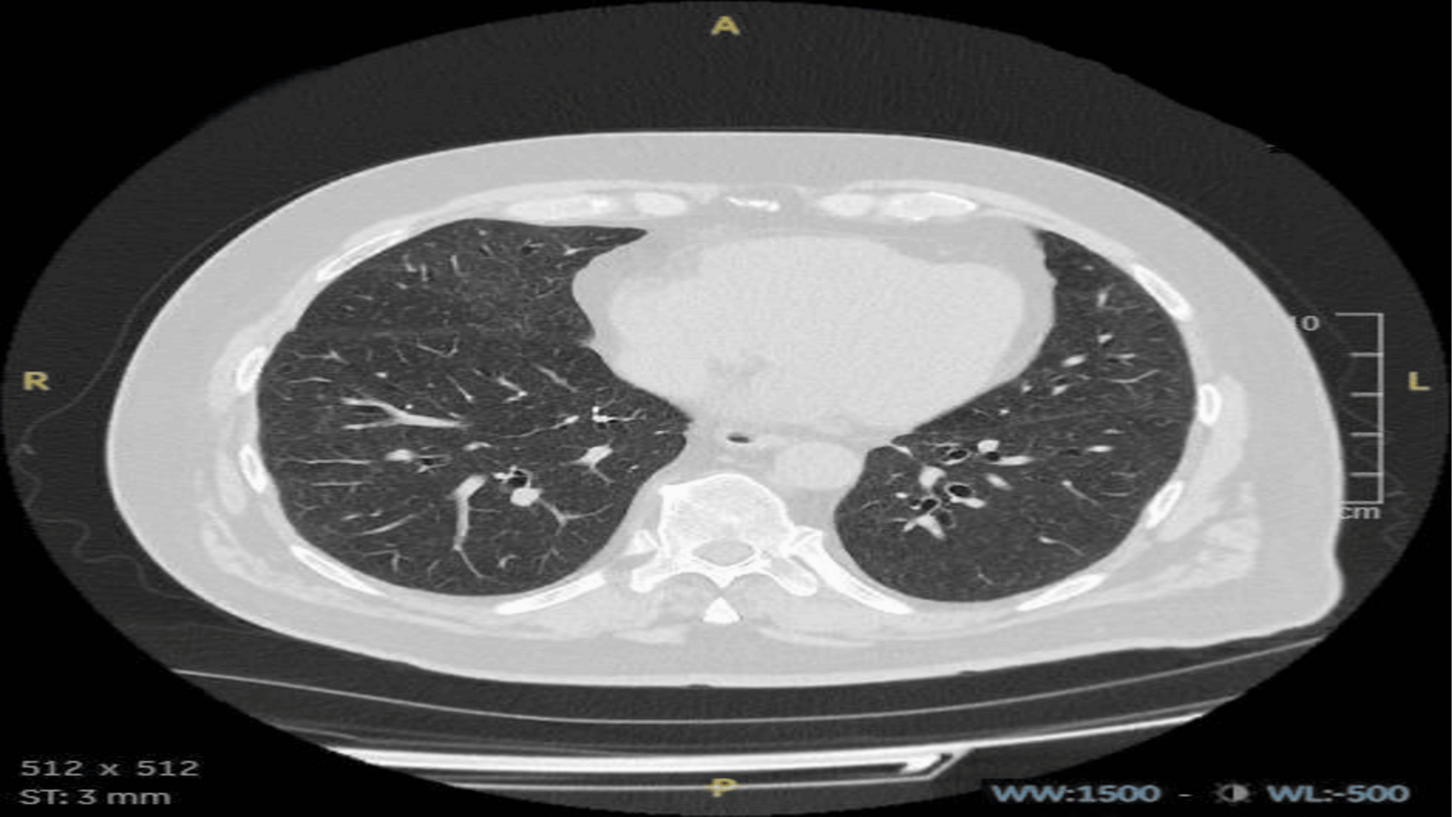
Repeat chest CT three months post-admission Repeat chest CT performed three months post-admission, following avoidance of hot tub exposure, showing significant resolution of previously observed ground-glass opacities and improving nodularity. CT, computed tomography

Between his discharge and the follow-up CT, the patient abstained from using the hot tub and remained symptom-free. Pulmonary function tests showed that the FVC, FEV1, FEV1/FVC ratio, and FEF25-75% were all within normal limits (Table 2, Figure 4). The repeat chest CT demonstrated significant resolution of the previously observed ground-glass opacities with resolving nodularity (Figure 5).

**Table 2:** Pulmonary function test results two months post-admission FEV1, forced expiratory volume in one second; FVC, forced vital capacity; PEF, peak expiratory flow; FEF, forced expiratory flow; SVC, slow vital capacity; FIF, forced inspiratory flow; IC, inspiratory capacity; ERV, expiratory reserve volume; DLCOunc, diffusion capacity uncorrected; DLCOcor, diffusing capacity for carbon monoxide corrected; DL, diffusing capacity; VA, alveolar volume

		Pre-bronchodilator		
	Actual	Predicted value (Pred)	% Pred	Z score
SPIROMETRY				
FEV1 (L)	2.92	2.91	100	+0.00
FVC (L)	3.25	3.73	87	-0.79
FEV1/FVC (%)	90	76	118	+1.98
PEF (L/min)	470.6			
FEF, 50% (L/sec)	5.79	4.06	142	+1.31
FEF 25-75% (L/sec)	4.69	3.19	146	+1.44
FEV1/SVC (%)	86	78	109	
FIF Max (L/sec)	3.74			
LUNG VOLUMES				
SVC (L)	3.41	3.73	91	-0.53
IC (L)	2.17	3.00	72	
ERV (L)	1.23	0.73	168	
DIFFUSION				
DLCOunc (mM/min/kPa)	6.18	9.35	66	
DLCOcor for hemoglobin (mM/min/kPa)		9.35		
DL/VA (mM/min/kPa/L)	1.34	1.45	92	
VA (L)	4.61	6.46	71	-2.65

**Figure 6:**
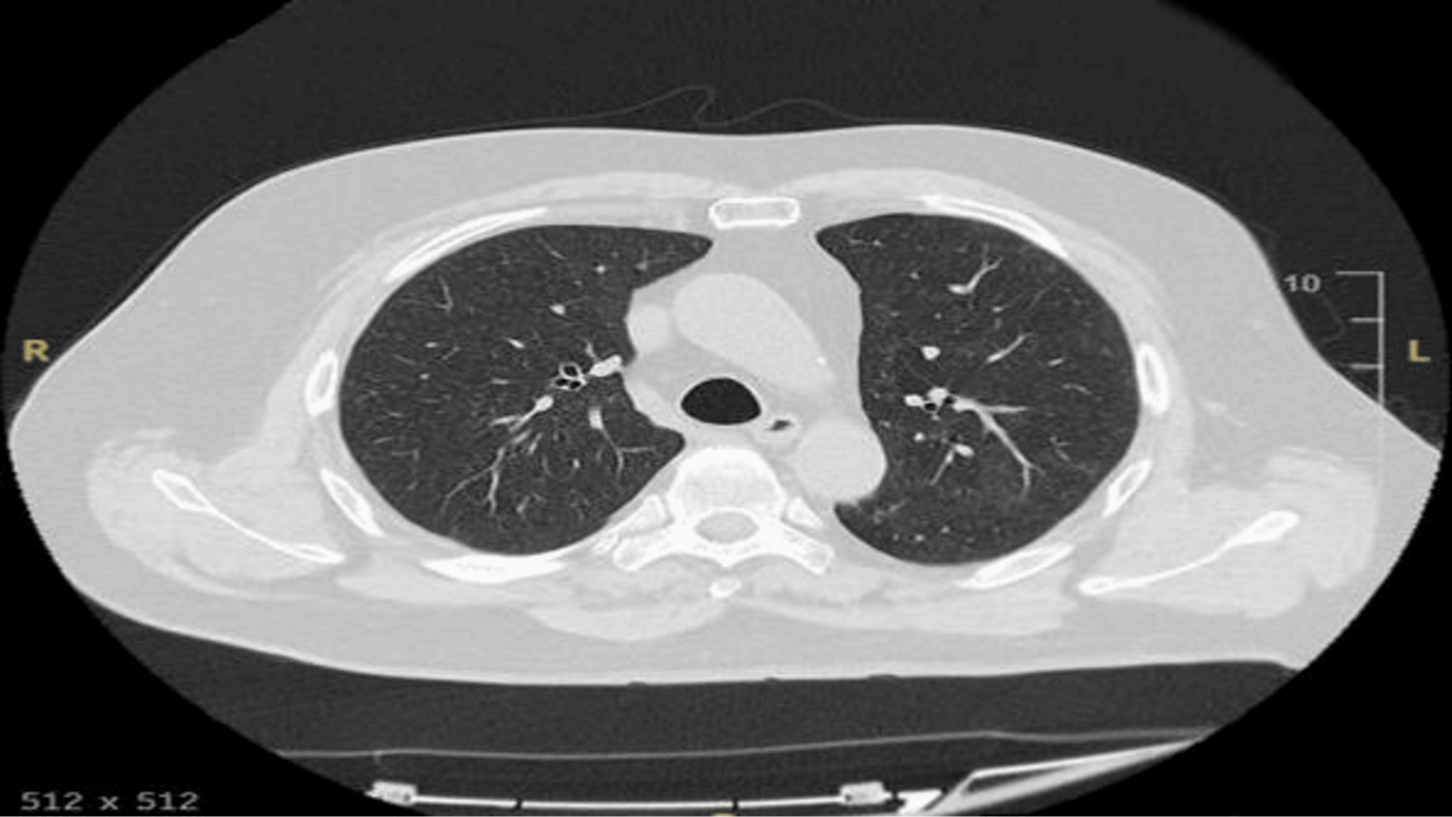
CT chest from one year ago: cancer surveillance follow-up The patient had a CT chest performed one year prior as part of a cancer surveillance follow-up, which was reported as normal. CT, computed tomography

In light of the above, the most likely diagnosis was determined to be either Chlorine-induced bronchiolitis obliterans or chlorine-induced HP-like reaction. Furthermore, a CT chest scan from a year ago, performed as part of cancer surveillance, was normal (Figure 6), which further supported this diagnosis.

## Discussion

Chlorine, a dense and irritating gas with a strong smell, mainly affects the lower respiratory tract and is commonly used for pool disinfection. Chlorine inhalation can damage both the airways and alveoli, with high levels leading to symptoms such as difficulty breathing, airway obstruction, coughing, cyanosis, nausea, vomiting, and loss of consciousness. The severity of the damage depends on the concentration: lower levels typically affect the airways, while higher concentrations can also harm the alveoli and cause pulmonary edema [1]. Children, competitive swimmers, and indoor pool employees are at increased risk of respiratory problems like bronchial hyperreactivity, asthma, and rhinitis due to their frequent chlorine exposure [3]. Chronic exposure to chlorine can result in bronchiolitis obliterans, a condition resulting from epithelial damage to the bronchioles [1].

When chlorine comes into contact with water, it reacts to form toxic byproducts, including HCl and HOCl, both of which are harmful to the airways. Inhalation of chlorine gas results in its hydration, leading to the production of HCl and HOCl, which can then interact with and damage the airway lining. Additionally, reactive oxygen species (ROS) such as superoxide (O_^2^_−), hydrogen peroxide (H_2_O_2_), and hydroxyl radicals may be generated by activated neutrophils and secondary mitochondrial dysfunction. Neutrophil myeloperoxidase can further convert hydrogen peroxide into more HOCl, exacerbating the injury to the airways [4]. 

Exposure to low concentrations of chlorine gas (up to 2 ppm) causes mucous membrane irritation. Higher exposures, between 9 ppm and 50 ppm, may lead to chemical pneumonitis and bronchiolitis obliterans. In animal studies, 200 ppm causes severe bronchial constriction, while 800 ppm is lethal to 50% of exposed animals. At 2000 ppm, immediate respiratory arrest occurs [4].

In bronchiolitis obliterans, chest radiography often appears normal or shows nonspecific signs like hyperinflation, peripheral vascular attenuation, and sometimes nodular opacities. Over time, lung volumes may increase and HRCT is more revealing, typically showing mosaic attenuation patterns, air-trapping, and peripheral bronchiolectasis [5,6].

HP is an interstitial lung disease characterized by a complex immune response in the lung parenchyma triggered by repeated inhalation of specific allergens to which the lungs are sensitized. HP commonly manifests with symptoms such as fever, malaise, cough, and shortness of breath shortly after significant exposure to an offending antigen. These symptoms typically subside quickly after eliminating exposure. However, some patients may continue to experience persistent shortness of breath, general discomfort, and weight loss [7]. The condition arises from the prolonged and repeated inhalation of various organic dusts or other substances, especially those of animal or vegetable origin, with chemicals being less frequently involved. Over 300 potential triggers have been identified, including bacteria, fungi, animal and plant proteins, low molecular weight chemicals, and aerosolized metal working fluid [8].

HTL is a differential of HP that occurs in individuals exposed to aerosols from hot tubs contaminated with MAC. While there is a recognized association between nontuberculous Mycobacteria (NTM)-HP and exposure to *Mycobacterium fortuitum* and *Mycobacterium phocaicum*, this is relatively rare [9]. Key risk factors for developing HTL include inadequate maintenance of hot tubs, poor ventilation, and excessive aerosolization [10]. Despite being triggered by infectious agents. It is important to note that not everyone exposed to MAC develops HTL, suggesting that those who do may have an underlying genetic predisposition to the condition [11].

HTL may initially present with flu-like symptoms, including cough, fever, and joint pain, which are later followed by persistent symptoms such as shortness of breath with exertion, fatigue, and weight loss [12]. Diagnosing HTL can be complex and requires a detailed evaluation of patient history, imaging findings, and pathological and microbiological evidence. The diagnostic criteria for HTL are (1) persistent respiratory symptoms; (2) diffuse lung infiltrates seen on chest X-rays or CT scans; (3) exposure to a hot tub prior to symptom onset; (4) detection of MAC in respiratory samples, hot tub water, or lung tissue biopsies; and (5) ruling out other potential causes for the condition [13].

HP is classified into nonfibrotic (NFHP) and fibrotic (FHP) forms. NFHP is an inflammatory condition marked by ground-glass opacities, mosaic attenuation, and signs of small airway disease, such as centrilobular nodules or air trapping. Mosaic attenuation reflects pneumonitis alongside normal or reduced attenuation due to bronchiolar obstruction. In the right context, ground-glass opacities, airspace consolidation, or lung cysts can also indicate NFHP. FHP, characterized by both fibrosis and bronchiolar obstruction, shows fibrosis in mid/lower lung zones with basilar sparing and features like ground-glass opacities, air trapping, or a three-density pattern. Fibrosis without bronchiolar obstruction is indeterminate for FHP [14]. In our case, the CT scan also revealed diffuse ground-glass changes with a centrilobular pattern in both lungs.

To manage HTL, the primary recommendation is to stop using hot tubs, as this typically leads to symptom relief. If symptoms persist, oral corticosteroids are the next step in treatment. For patients who test positive for MAC or NTM, anti-mycobacterial drugs may be considered if the corticosteroids are not effective, though the optimal duration of this treatment and the specific patient criteria remain unclear [15].

There is no specific treatment for injuries caused by chlorine gas inhalation; management primarily involves supportive care. Bronchodilators are recommended for patients with bronchospasm or wheezing, and corticosteroids may also be considered. Avoiding further exposure to chlorine gas is advised [16]. In our patient, recovery was achieved through avoidance of the hot tub and the use of oral steroids.

This case illustrates the diagnostic challenges when respiratory symptoms, CT findings, and a history of hot tub use initially suggest HTL, typically caused by inhaling MAC from contaminated water. Despite the initial suspicion of HTL, the absence of Mycobacterium species in diagnostic tests, along with the patient’s exposure to high chlorine levels in the hot tub, prompted a reconsideration of the diagnosis. The improvement in symptoms and imaging on repeat scans further supported the revised diagnosis. It was ultimately determined that the patient may have had chlorine-induced bronchiolitis obliterans or chlorine-induced HP, both conditions that can result from exposure to high chlorine levels. The lack of a lung biopsy leaves some uncertainty, but the clinical course and exposure history strongly suggest a chlorine-related lung injury.

## Conclusions

This case underscores the diagnostic complexities of distinguishing between HTL and chlorine-induced lung injuries. Despite the lack of a lung biopsy, the clinical evidence supports a diagnosis of chlorine-related lung injury, highlighting the need for careful consideration of exposure history and diagnostic criteria in respiratory conditions.
